# Impact of Insomnia on Myocardial Infarction Risk and Coronary Plaque Vulnerability: Insights From Mendelian Randomization and OCT Imaging

**DOI:** 10.1002/clc.70226

**Published:** 2025-12-02

**Authors:** Yang Gao, Qingbo Shi, Zhuocheng Shi, Zhiwen Zhang, Haosen Yu, Mingxing Lv, Tong Zhang, Donghui Chen, Yu shuo Gu, Quan Guo, Muwei Li, Cao Ma

**Affiliations:** ^1^ Zhengzhou University People's Hospital, Central China Fuwai Hospital of Zhengzhou University, Fuwai Central China Cardiovascular Hospital Zhengzhou China; ^2^ Central China Subcenter of National Center for Cardiovascular Diseases, Henan Cardiovascular Disease Center Zhengzhou China

**Keywords:** insomnia, Mendelian randomization, myocardial infarction, optical coherence tomography, thin‐cap fibroatheroma

## Abstract

**Background:**

Insomnia is a prevalent sleep disorder increasingly recognized as a risk factor for cardiovascular diseases (CVDs). However, its causal relationship with myocardial infarction (MI) and its impact on coronary plaque vulnerability remain poorly understood.

**Methods:**

We performed Mendelian randomization (MR) analysis using genome‐wide association study (GWAS) summary data for insomnia (*n* = 462,341) and MI (*n* = 484,598) in populations of European descent. Additionally, 340 patients with coronary artery disease (CAD) underwent coronary angiography and optical coherence tomography (OCT) imaging. Insomnia was assessed by the Insomnia Severity Index (ISI), and OCT was used to evaluate plaque features including thin‐cap fibroatheroma (TCFA), fibrous cap thickness, lipid arc, macrophage infiltration, and plaque rupture.

**Results:**

MR analysis showed a potential causal effect of genetically predicted insomnia on MI risk (OR = 1.015; 95% CI: 1.004–1.027; *p* = 0.007), with no evidence of pleiotropy or heterogeneity. Clinically patients with insomnia (ISI ≥ 8) had higher rates of hypertension (54.3% vs. 39.6%) and MI (32.4% vs. 21.7%), elevated CRP levels, and exhibited greater plaque vulnerability on OCT, including increased incidence of TCFA (29.5% vs. 17.0%), thinner fibrous caps, larger lipid arcs, and more frequent macrophage infiltration and plaque rupture. Logistic regression identified both insomnia (OR = 1.806; *p* = 0.037) and CRP (OR = 1.384; *p* = 0.034) as independent predictors of TCFA.

**Conclusions:**

This study provides genetic and clinical evidence that insomnia contributes to MI risk and coronary plaque vulnerability, underscoring the importance of addressing sleep disturbances in CAD management.

## Graphical abstract

This integrated study demonstrates that insomnia contributes to myocardial infarction (MI) risk and coronary plaque vulnerability. Leveraging genetic data (*n* = 462,341), Mendelian randomization established a potential causal effect of insomnia on MI (OR = 1.015, *p* = 0.007). Clinically, in patients with coronary artery disease (n = 340), insomnia was independently associated with thin‐cap fibroatheroma (TCFA), a key feature of vulnerable plaque (OR = 1.806, *p* = 0.037). These findings underscore the importance of addressing sleep disturbances in cardiovascular risk management.

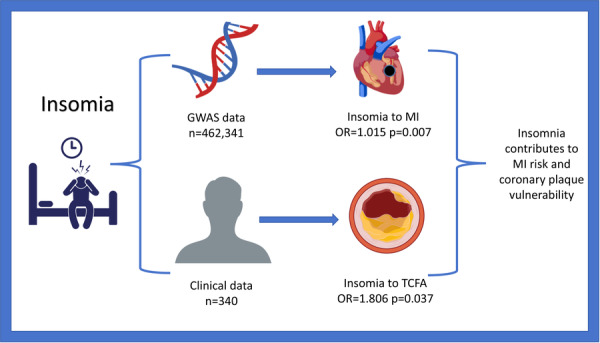

## 1 Background

Insomnia is one of the most common sleep disorders, affecting approximately 20% of the global population and up to 50% of patients in primary care. It often coexists with medical or psychiatric conditions and, if left untreated, can contribute to their onset or progression. Characterized by difficulty initiating or maintaining sleep along with daytime dysfunction, insomnia is increasingly recognized as a risk factor for adverse health outcomes [1]. In the past decade, numerous observational studies have demonstrated an association between insomnia and increased cardiovascular disease (CVD) morbidity and mortality. Building on this, a systematic meta‐review further support potential causal links between insomnia and several CVDs, including coronary artery disease (OR = 1.14), hypertension (OR = 1.16), atrial fibrillation (OR = 1.02), heart failure (OR = 1.04), and large artery stroke (OR = 1.14). These findings reinforce insomnia as an independent risk factor and a promising target for CVD prevention [2, 3]. Coronary vulnerable plaques typically exhibit a large lipid core, a thin fibrous cap, and infiltration of macrophages. Thin‐cap fibroatheroma (TCFA) represents the prototypical manifestation of vulnerable plaques and is regarded as a precursor to plaque rupture, which may lead to myocardial infarction(MI) [4]. While intravascular optical coherence tomography (OCT) has emerged as the gold standard for TCFA identification, no studies to date have investigated the potential relationship between insomnia and TCFA formation [5, 6].

To address this knowledge gap, we first employed Mendelian randomization (MR) analysis to establish genetic evidence for insomnia's causal role in MI pathogenesis. Building on these findings, we then conducted detailed OCT evaluations to examine the association between insomnia and coronary plaque vulnerability in patients with coronary artery disease (CAD). This dual‐method approach not only elucidates insomnia's cardiovascular impact at both genetic and phenotypic levels but also underscores the critical importance of sleep management in CAD patient care.

## 2 Methods

### 2.1 Data Sources and Patient Population

The MR Study data derived from the IEU OpenGWAS database (https://gwas.mrcieu.ac.uk/). To decrease possible bias from stratification of the population, MR analyses were limited to people of European descent. For the data set of insomnia, we used summary statistics from a GWAS of UK Biobank with a sample size of 462,341 (ukb‐b‐3957). We chose MI with a sample size of 484,598 as outcome (ebi‐a‐GCST90038610).

To further evaluate the relationship between insomnia and myocardial infarction, a total of 358 consecutive patients (age ≥ 18 years) with coronary heart disease who underwent coronary angiography and OCT in Central China Fuwai Hospital of Zhengzhou University from January 2021 to January 2023 were enrolled. After the examination, an experienced investigator administered a questionnaire to all patients to assess their sleep status.

Inclusion criteria: (1) meet the American Heart Association diagnostic criteria for CAD [7], (2) complete coronary angiography and OCT examination.

Exclusion criteria: (1) OCT image quality was poor and could not be analyzed; (2) patients are unable to cooperate with the questionnaire.

**Figure 1:**
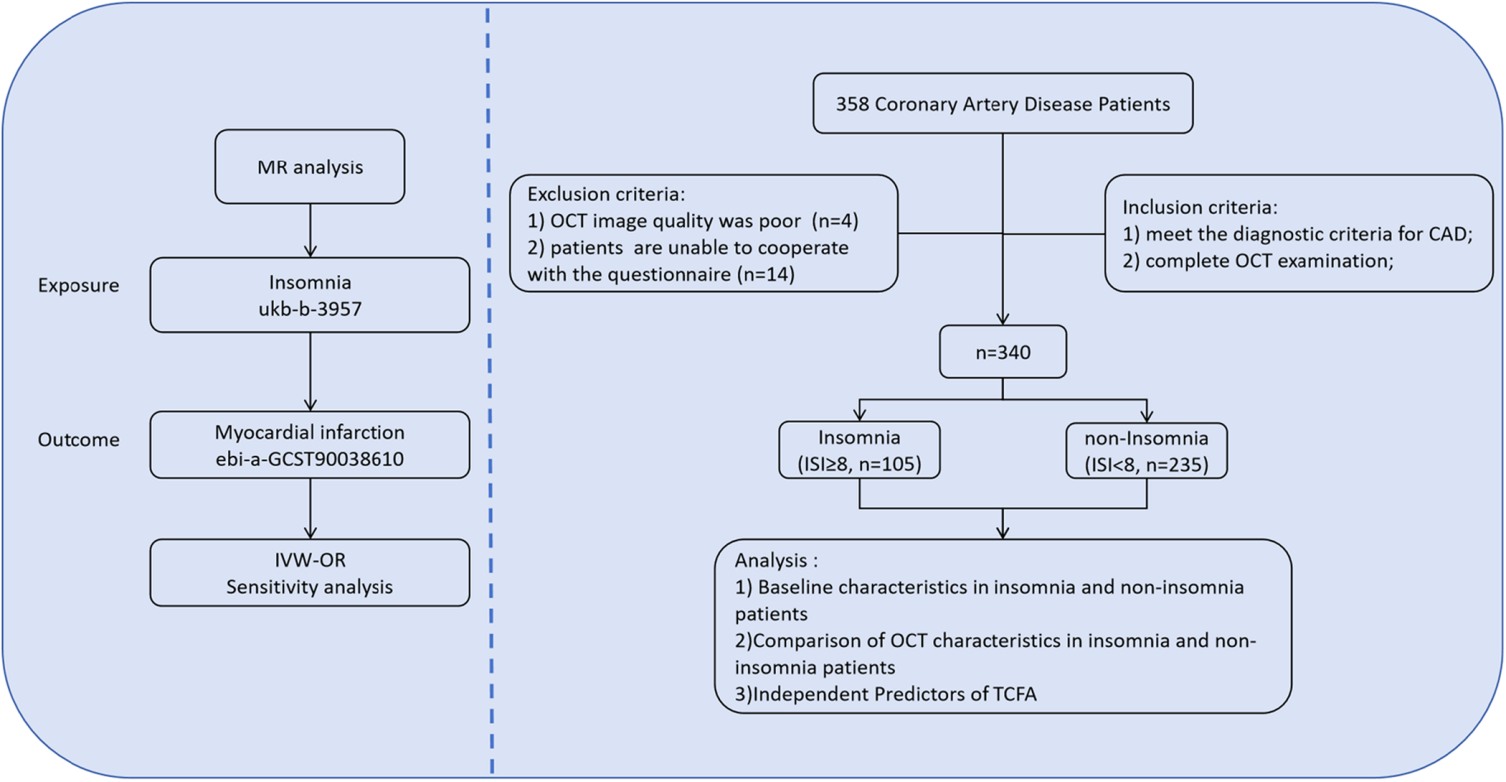
**F**low chart of the study.

Following the Declaration of Helsinki (as revised in 2013), this study was approved by the Medical Ethics Committee of Central China Fuwai Hospital of Zhengzhou University. Informed consent was obtained from all patients, and the data collected were kept confidential, in accordance with ethical standards. The flow chart of this study is shown in Figure 1.

### 2.2 Selection of IVs

This study satisfied the three assumptions of classical MR Analysis: 1. IVs directly affected exposure; 2. IVs were not associated with confounders; and 3. IVs influenced the risk of outcomes directly through exposure, not through other pathways [8]. To meet these conditions, we selected SNPs associated with exposure factors (*P* < 5 × 10^−8^) as candidate IVs firstly. Independent instrumental variables (IVs) were obtained by excluding SNPs in linkage disequilibrium (LD) with a threshold of *r* ^2^ = 0.001 and kb=10,000, secondly. To excluded potential pleiotropic effects, “Phenoscanner V2” was used to remove SNPs that corresponding to the phenotype related to the outcome thirdly [9]. Finally, we used F‐statistics to evaluate the strength of the IVs and avoid weak instrument bias [10]. We selected SNPs with *F* > 10, considered a valid threshold, as instrumental variables. The F statistic was calculated using the formula *F* = *β* ^2^/*SE* ^*2*^, where *β* represents the effect on the risk of exposure and SE is the standard error.

### 2.3 Evaluation of Insomnia

The insomnia of the enrolled patients was investigated by trained investigators using Insomnia Severity Index (ISI). The ISI is a 7‐item self‐report questionnaire assessing the nature, severity, and impact of insomnia. The usual recall period is the “last month” and the dimensions evaluated are: severity of sleep onset, sleep maintenance, and early morning awakening problems, sleep dissatisfaction, interference of sleep difficulties with daytime functioning, noticeability of sleep problems by others, and distress caused by the sleep difficulties. A 5‐point Likert scale is used to rate each item (e.g., 0 = no problem; 4 = very severe problem), yielding a total score ranging from 0 to 28. The total score is interpreted as follows: absence of insomnia (0–7); sub‐threshold insomnia (8–14); moderate insomnia (15–21); and severe insomnia (22–28) [11]. Based on previous study, we define insomnia as ISI ≥ 8 [12].

### 2.4 OCT Imaging

Two experienced doctors completed the OCT examination of the culprit vessels by non‐balloon occlusion technique. The OCT imaging catheter was sent to the distal end of the culprit vessel along the guide wire. After the injection of contrast agent, the OCT imaging catheter was automatically pulled back at a retraction speed of 18 mm/s and a rotation speed of 180 frames/s. The OCT imaging catheter was withdrawn at the end of imaging. All OCT procedures were performed after coronary arteriography was completed, and intracoronary nitroglycerin was administered before the OCT examination.

### 2.5 OCT Analysis

**Figure 2:**
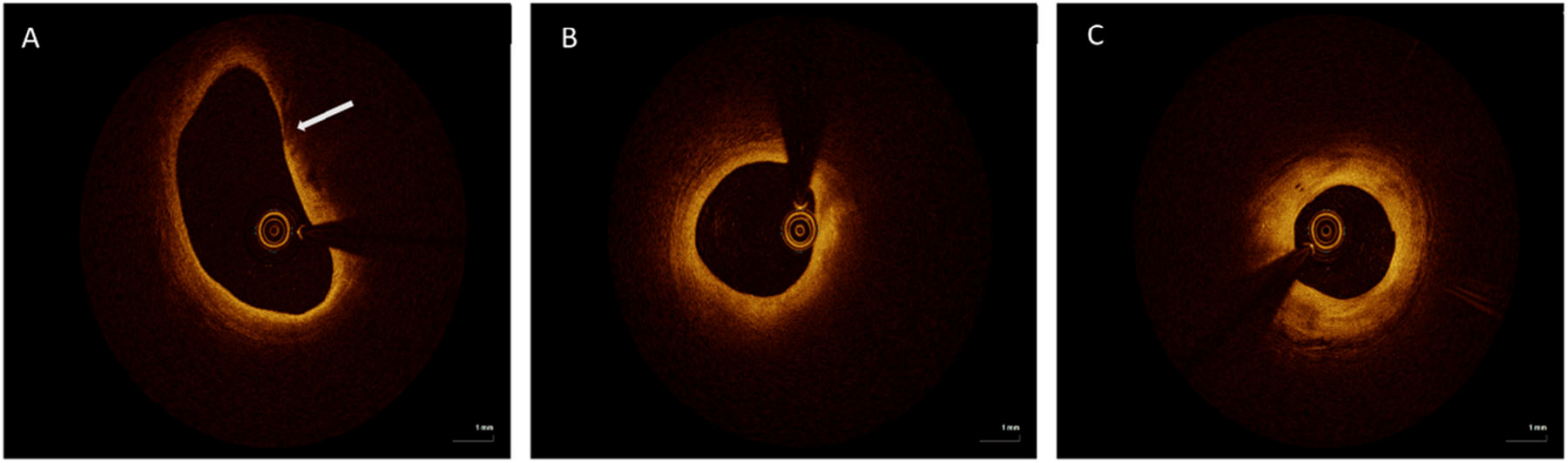
Characteristics of plaques under OCT. (A): thin‐cap fibroatheroma (TCFA) (B): non‐TCFA (C): fibrous plaque.

OCT images of all patients were analyzed by two independent and experienced interventional technicians who were unaware of the patients' clinical information. In case of inconsistent observations, concordant results were obtained from a third interventional technician. OCT images were analyzed according to the consensus standards for acquisition, measurement, and reporting of intravascular optical coherence tomography studies [5]. OCT was performed before any intervention (e.g., balloon predilation). Plaques were defined as ≥ 1 mm axial length with intimal thickening exceeding adjacent reference segments by ≥ 0.5 mm. The culprit vessel plaque length, distal reference area, proximal reference area, minimum lumen area, fibrous cap thickness, lipid arc, TCFA, macrophage, cholesterol crystal, microchannel, calcified nodule, plaque erosion and other data were analyzed. The fibrous cap thickness covering the lipid core was measured in triplicate at its thinnmost part, and the mean of the three measurements was calculated. Lipid arc were measured at 1 mm intervals throughout the lesion and maximal radians were recorded. TCFA is defined as a lipid‐rich plaque with a maximum lipid arc ≥ 90° and a fibrous cap thickness of ≤ 65 μm [5, 13]. Figure 2

### 2.6 Statistical Analysis

Five different methods of MR analyses, i.e., the MR‐Egger, WM, inverse variance weighted (IVW), simple mode, and weighted mode were implemented. The IVW method was used as the principal MR analytic approach to assess the associations between insomnia and the risk of MI. All statistical analyses were performed using R software (version 4.2.3). Analyses were performed using TwoSampleMR R package, which included IVW, Mendelian Randomization‐Egger (MR‐Egger), weighted median, simple mode, and weighted mode. We used the IVW (Q) and MR‐Egger regression (Q) methods to test for heterogeneity, the MR‐Egger intercept test to evaluate the horizontal pleiotropy [14], and the leave‐one‐out analysis to exclude the possible influence of individual SNPs on the overall results.

The clinical information of the patients was statistically analyzed using SPSS 26.0 statistical software (SPSS Inc, Chicago, IL, USA). The normality of all continuous variables was formally assessed using the Shapiro‐Wilk test. Since the majority of variables significantly deviated from a normal distribution (*p* < 0.05), all continuous data are presented as median (interquartile range, IQR). Accordingly, comparisons between two groups were performed using the Mann‐Whitney U test. Categorical data are presented as numbers (%) and compared using the chi‐square test. The Cochran‐Armitage trend test was used to assess the presence of a linear trend in proportions across ordered groups. Spearman rank‐order correlation was used to assess associations between ordinal insomnia and vulnerable plaque features, with *p* < 0.05 considered statistically significant. Variables with a *p*‐value < 0.05 in the univariate analysis were included in the binary logistic regression analysis using the enter method. Variance Inflation Factor (VIF) was applied to assess multicollinearity, with a VIF > 10 used as the threshold indicating severe multicollinearity. Model fit was evaluated using the Hosmer‐Lemeshow test (*p* > 0.05). All statistical analyses were performed using bilateral tests. The statistical significance was set at *p* < 0.05.

## 3 Results

### 3.1 MR Analysis Results of Insomnia to Myocardial Infarction

We extracted IVs that were significantly related to insomnia from the GWAS (*P* < 5 × 10^−8^) and removed LD (*r* ^2^ < 0.001, 10,000‐kb). The SNPs that were associated with confounding factors and SNPs for palindromic or incompatible alleles were removed. Finally, 39 independent insomnia‐associated SNPs were obtained as IVs. The screened SNPs were included in further analyses. No evidence of weak‐tool bias was found in the IVs strength test (*F*‐statistic> 10). Using the IVW method, we found that genetically insomnia might have potential relationship with higher odds of MI [IVW: OR, 1.015; 95% CI, 1.004, 1.027; *p* = 0.007]. The results of the analysis are shown in Table 1.

**Table 1:** MR analyses between insomnia to MI.

Exposure	Outcome	Methods	SNP numbers	*β*	SE	*P*	OR	95% CI
Insomnia	MI	MR Egger	39	0.006	0.018	0.763	1.006	0.970, 1.042
Insomnia	MI	Weighted median	39	0.013	0.008	0.092	1.013	0.998, 1.029
Insomnia	MI	IVW	39	0.015	0.006	0.007	1.015	1.004, 1.027
Insomnia	MI	Simple mode	39	0.018	0.016	0.263	1.018	0.987, 1.051
Insomnia	MI	Weighted mode	39	0.015	0.012	0.221	1.016	0.991, 1.041

Abbreviations: β, beta; CI, confidence interval; IVW, inverse‐variance weighted; MR, Mendelian randomization; MI, myocardial infarction; OR, odds ratio; SNP, single‐nucleotide polymorphism; SE, standard error.

### 3.2 Sensitivity and Robustness Analyses

**Figure 3:**
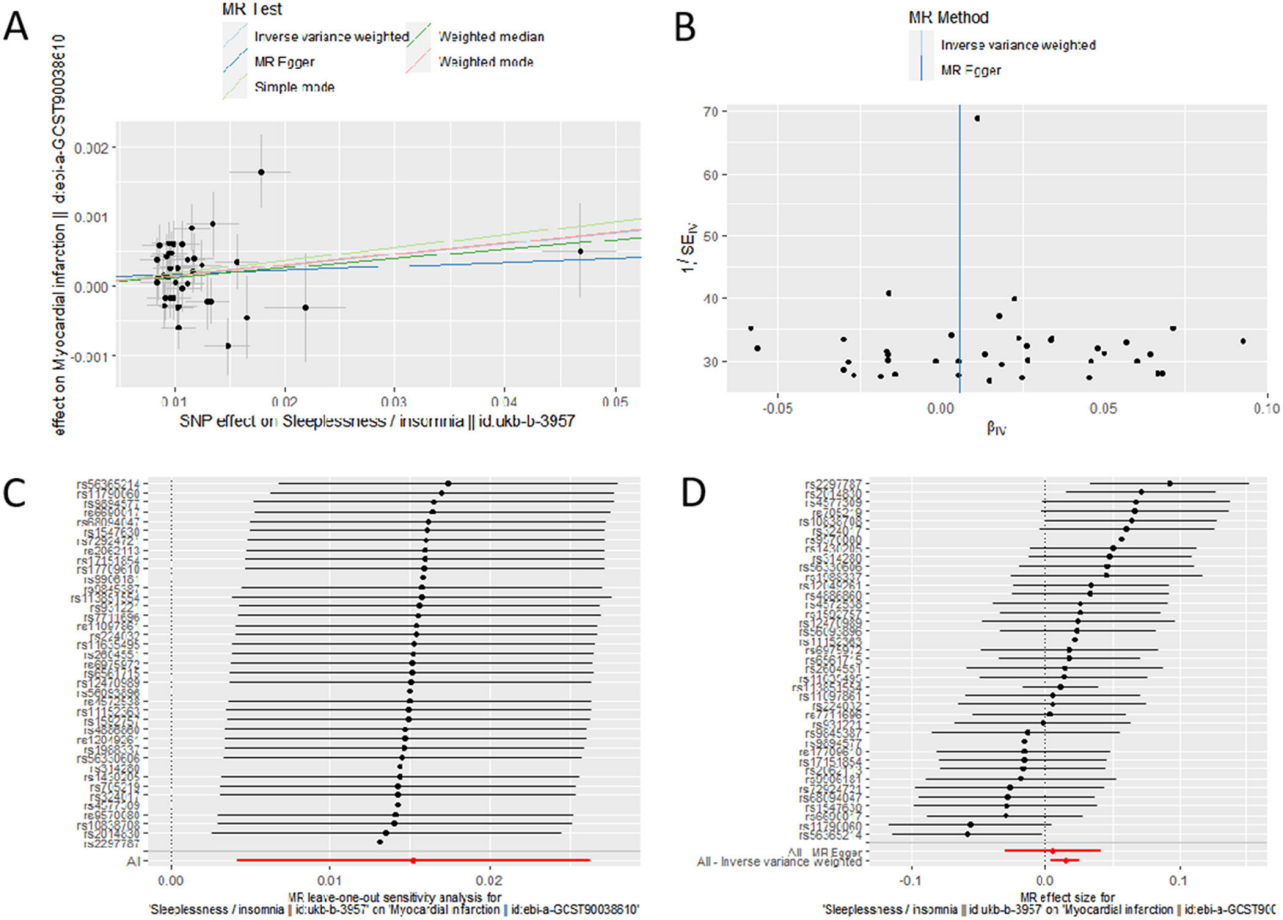
Scatter plot, Funnel plot, Leave‐one‐out method plot and forest plot of insomnia to MI (A), Scatter plot; (B), Funnel plot; (C), Leave‐one‐out method plot; (D), forest plot).

The IVW (Q), MR‐Egger (Q), and MR‐Egger intercept tests were performed as sensitivity analyses to detect the potential heterogeneity and horizontal pleiotropy and there showed no pleiotropy and heterogeneity. The results of the analysis are shown in Table 2. In addition, the leave‐one‐out method revealed that the potential causal correlation between insomnia and MI was not driven by a single SNP. Funnel and forest plots, which could more intuitively show heterogeneity. The results of the analysis are shown in Figure 3.

**Table 2:** Pleiotropy and heterogeneity test of the insomnia IVs to MI.

Outcomes	Heterogeneity test						
	MR‐Egger				Inver‐variance weighted		
	Q	Q_df	Q_*p*val		Q	Q_df	Q_*p*val
MI	51.080	37	0.062		51.504	38	0.071
	Pleiotropy test						
	MR‐Egger						
	Intercept		SE			*P*	
	1.15E‐04		2.07E‐04			0.583	

Abbreviations: df, degree of freedom; MI, myocardial infarction; MR, Mendelian randomization; Q, heterogeneity statistic Q.

### 3.3 Baseline Characteristics in Insomnia and Non‐Insomnia Patients

A total of 358 consecutive patients with CAD who underwent coronary angiography and OCT in Central China Fuwai Hospital of Zhengzhou University from January 2021 to January 2023 were enrolled. 18 patients were excluded due to poor image quality (*n* = 14) and unable to cooperate with the questionnaire (*n* = 4). The patients were divided into insomnia group (n = 105) and non‐insomnia group (*n* = 235) according to the ISI in the final analysis. Table 3 shows baseline clinical characteristics for both groups. In the baseline characteristics, both the prevalence of hypertension (54.29% vs. 39.57%, *p* < 0.05) and MI (32.38% vs. 21.70%, *p* < 0.05) were higher in insomnia group than non‐insomnia group. Laboratory examination showed that the level of CRP in insomnia group was significantly higher than non‐insomnia group [2.89 (2.22,3.39) vs. 2.42 (1.81,3.19), *p* < 0.05].

**Table 3:** Baseline characteristics in insomnia and non‐insomnia patients.

Variable	Non‐insomnia (*n* = 235)	Insomnia (*n* = 105)	Test values	*p* value
Age, years	57.00 (49.00, 67.00)	58.00 (48.00, 69.00)	−0.063	0.950
Male, *n* (%)	187 (79.57)	78 (74.29)	1.181	0.277
BMI, kg/m^2^	25.80 (24.30, 27.70)	25.30 (23.55, 27.10)	−1.572	0.116
Family history, *n* (%)	30 (12.77)	9 (8.57)	1.257	0.262
Diabetes, *n* (%)	68 (28.94)	36 (34.29)	0.978	0.323
Hypertension, *n* (%)	93 (39.57)	57 (54.29)	6.371	0.012
Smoking history, *n* (%)	115 (48.94)	49 (55.24)	0.150	0.699
Drinking history, *n* (%)	70 (29.79)	27 (25.71)	0.590	0.442
ACS, *n* (%)	126 (53.617)	64 (60.95)	1.584	0.208
MI, *n* (%)	51 (21.70)	34 (32.38)	4.414	0.036
eGFR, ml/min/1.73m^2^	89.23 (81.31, 99.22)	91.13 (82.72, 102.42)	−1.166	0.244
AST, U/L	20.60 (15.00, 26.10)	19.00 (15.95, 24.65)	−0.898	0.369
ALT, U/L	26.80 (19.00, 33.80)	25.80 (16.35, 32.05)	−1.198	0.231
TC, mmol/L	3.33 (2.86, 3.90)	3.50 (3.00, 4.01)	−1.133	0.257
TG, mmol/L	1.32 (0.98, 1.81)	1.47 (1.05, 2.01)	−1.429	0.153
LDL‐C, mmol/L	2.09 (1.73, 2.52)	2.22 (1.77, 2.71)	−1.160	0.246
HDL‐C, mmol/L	0.99 (0.85, 1.20)	0.99 (0.87, 1.12)	−0.572	0.567
HbA1c, %	5.75 (5.41, 6.18)	5.86 (5.56, 6.21)	−1.279	0.201
Glucose, mmol/L	5.03 (4.54, 5.94)	5.30 (4.66, 6.22)	−1.388	0.165
CRP, mg/L	2.42 (1.81, 3.19)	2.89 (2.22, 3.39)	−2.904	0.004
Antihypertensive drugs, *n* (%)	91 (38.70)	54 (51.40)	4.790	0.029
Lipid‐lowering drugs, *n* (%)	124 (86.00)	56 (82.90)	0.547	0.459
Antidiabetic drugs, *n* (%)	66 (28.10)	34 (32.40)	0.645	0.422

Abbreviations: ACS, acute coronary syndrome; ALT, alanine transaminase; AST, aspartate aminotransferase; BMI, body mass index; CRP, C‐reactive protein; eGFR, estimated glomerular filtration rate; HDL‐C, high‐density lipoprotein cholesterol; HbA1c, glycated hemoglobin; LDL‐C, low‐density lipoprotein cholesterol; MI, myocardial infarction; TC, total cholesterol; TG, triglycerides.

### 3.4 Comparison of OCT Characteristics in Insomnia and Non‐Insomnia Patients

A comparative analysis of plaque characteristics between the two groups, as evaluated by optical coherence tomography, is presented in Table 4. Compared with non‐insomnia group, the insomnia group had a significantly higher incidence of TCFA(29.52% vs. 17.02%, *p* < 0.05) and a significantly thinner fiber cap thickness [62.00 (48.50,209.00) vs. 94.00 (62.00,192.00), *p* < 0.05]. In insomnia group, lipid arc [220.00 (180.00,295.00) vs. 180.00 (150.00,270.00), *p* < 0.05] and the incidence of macrophage (38.10% vs. 22.55%, *p* < 0.05) and plaque rupture (32.28% vs. 21.28%, *p* < 0.05) were also higher than that in non‐insomnia group.

**Table 4:** Angiographic and OCT characteristics in insomnia and non‐insomnia patients.

Variable	Non‐insomnia (*n* = 235)	Insomnia (*n* = 105)	Test values	*p* value
Culprit vessels, *n* (%)			0.631	0.729
LAD	172 (73.19)	76 (72.38)		
LCX	25 (10.64)	14 (13.33)		
RCA	38 (16.17)	15 (14.29)		
Plaque length, mm	21.87 (16.47, 26.84)	23.48 (17.09, 28.92)	−0.914	0.361
Distal reference diameter, mm	3.10 (2.83, 3.47)	3.15 (2.77, 3.56)	−0.517	0.605
Distal reference area, mm^2^	7.56 (6.33, 9.48)	7.83 (6.36, 9.95)	−0.643	0.521
Proximal reference diameter, mm	3.50 (3.12, 3.85)	3.53 (3.14, 3.84)	−0.562	0.574
Proximal reference area, mm^2^	9.57 (7.65, 11.63)	9.80 (7.76, 11.65)	−0.586	0.558
Minimum lumen area, mm^2^	2.61 (1.98, 3.09)	2.50 (1.82, 2.92)	−1.001	0.317
Fibrous cap thickness, μm	94.00 (62.00, 192.00)	62.00 (48.50, 209.00)	−2.098	0.036
Lipid arc, deg	180.00 (150.00, 270.00)	220.00(180.00, 295.00)	−4.381	< 0.001
TCFA, *n* (%)	40 (17.02)	31 (29.52)	6.866	0.009
Macrophage, *n* (%)	53 (22.55)	40 (38.10)	8.822	0.003
Cholesterol crystal, *n* (%)	84 (35.74)	36 (34.29)	0.068	0.795
Microchannel, *n* (%)	72 (30.64)	28 (26.67)	0.551	0.458
Calcified nodule, *n* (%)	19 (8.09)	3 (2.86)	3.278	0.070
Plaque rupture, *n* (%)	50 (21.28)	34 (32.38)	4.811	0.028
Plaque erosion, *n* (%)	52 (22.13)	27 (25.71)	0.661	0.416
Thrombus, *n* (%)	76 (32.34)	38 (36.19)	0.483	0.487

Abbreviations: LAD, left anterior descending artery; LCX, left circumflex artery; RCA, right coronary artery; TCFA, thin‐cap fibroatheroma

### 3.5 Independent Predictors of TCFA

Insomnia was weakly but significantly associated with thinner fibrous caps (r = −0.114, *p* = 0.036) and larger lipid arcs (r = 0.238, *p* < 0.001), suggesting a potential link with plaque instability. Baseline characteristics were reanalyzed according to TCFA presence, revealing statistically significant differences in insomnia, CRP, TC, and LDL‐C levels between the two groups (Table 5). There was a significant increasing trend in insomnia severity in the TCFA group compared with the non‐TCFA group (Cochran–Armitage trend test, Z = 2.12, *p* = 0.034). These variables, all of which showed no significant multicollinearity, were subsequently included in the logistic regression model to identify independent predictors of TCFA. As shown in Table 6, after adjusting for relevant covariates, both insomnia (OR = 1.806, 95% CI = 1.037–3.146, *p* < 0.05) and CRP (OR = 1.384, 95% CI = 1.026–1.867, *p* < 0.05) emerged as independent factors significantly associated with TCFA. The Hosmer‐Lemeshow goodness‐of‐fit test showed no significant deviation from the model (χ² =5.423, df = 8, *p* = 0.712), suggesting good calibration.

**Table 5:** Baseline characteristics in TCFA and non‐TCFA patients.

Variable	TCFA (*n* = 71)	non‐TCFA (*n* = 269)	Test values	*p* value
Age, years	58.00 (49.00, 69.00)	56.00 (48.50, 67.00)	−0.792	0.428
Male, *n* (%)	55 (77.46)	210 (78.07)	0.012	0.913
BMI, kg/m^2^	25.30 (23.60, 26.70)	25.80 (24.30, 27.70)	−1.859	0.063
Insomnia, *n* (%)	31 (43.66)	74 (27.51)	26.552	< 0.001
Mild insomnia	5 (7.04)	31 (11.52)		
Moderate insomnia	9 (12.68)	30 (11.15)		
Severe insomnia	17 (23.94)	13 (4.83)		
Family history, *n* (%)	6 (8.45)	33 (12.27)	0.806	0.369
Diabetes, *n* (%)	24 (33.80)	80 (29.74)	0.437	0.509
Hypertension, *n* (%)	32 (45.07)	118 (43.87)	0.033	0.856
Smoking history, *n* (%)	40 (56.34)	124 (46.10)	2.360	0.125
Drinking history, *n* (%)	20 (28.17)	77 (28.62)	0.006	0.940
eGFR, ml/min/1.73m^2^	88.35 (79.41, 99.11)	89.63 (82.51, 100.63)	−1.011	0.312
AST, U/L	20.70 (16.40, 26.05)	19.50 (15.00, 25.00)	−1.433	0.152
ALT, U/L	26.00 (21.15, 34.50)	26.70 (18.00, 33.00)	−0.716	0.474
TC, mmol/L	3.57 (3.17, 4.12)	3.34 (2.84, 3.88)	−2.065	0.039
TG, mmol/L	1.46 (1.06, 2.02)	1.32 (1.03, 1.83)	−1.320	0.187
LDL‐C, mmol/L	2.26 (1.92, 2.75)	2.06 (1.72, 2.49)	−2.428	0.015
HDL‐C, mmol/L	1.01 (0.91, 1.17)	0.99 (0.82, 1.17)	−1.243	0.214
HbA1c, %	5.74 (5.39, 6.09)	5.84 (5.49, 6.21)	−0.854	0.393
Glucose, mmol/L	5.05 (4.36, 5.85)	5.18 (4.61, 6.10)	−1.151	0.250
CRP, mg/L	3.02 (2.35, 3.44)	2.45 (1.842, 3.18)	−2.912	0.004
Antihypertensive drugs, *n* (%)	32 (45.10)	113 (42.00)	0.215	0.643
Lipid‐lowering drugs, *n* (%)	57 (80.30)	232 (86.20)	1.567	0.211
Antidiabetic drugs, *n* (%)	23 (32.40)	77 (28.60)	0.385	0.535

**Table 6:** Multivariate Regression Model for TCFA.

Variable	*β*	SE	Wald	OR	95% CI	*P*
Insomnia	0.591	0.283	4.365	1.806	1.037–3.146	0.037
CRP	0.325	0.153	9.700	1.384	1.026–1.867	0.034
TC	0.113	0.263	0.183	1.119	0.668–1.874	0.669
LDL‐C	0.264	0.303	0.759	1.302	0.719–2.360	0.384

Abbreviations: CRP, C‐reactive protein; TCFA, thin‐cap fibroatheroma.

## 4 Discussion

This study was divided into two parts, first, we discussed the relationship between insomnia and TCFA in CAD patients, and further studied the influence of insomnia on MI from the genetic perspective. The results showed that insomnia group has a higher proportion of MI (32.38% vs. 21.70%), and insomnia was an independent influencing factor for TCFA (*OR* = 2.397). The MR analysis revealed that insomnia might have potential relationship with higher odds of MI in genetics(*OR* = 1.015). Therefore, we suppose that insomnia may lead to an increased incidence of TCFA and further contribute to the occurrence of MI.

Insomnia is one of the most prevalent health concerns in the population and in clinical practice [15]. In a population‐based study of 52,610 adults followed for 11.4 years, researchers found that insomnia symptoms significantly increased the risk of MI, even after adjusting for cardiovascular risk factors [16]. Meta analysis revealed that insomnia was related to cardiovascular outcomes including atrial fibrillation (SRR: 1.30, 95% CI: 1.26 to 1.35), CVD (1.45, 1.29 to 1.64), CAD (1.28, 1.10 to 1.50), MI (1.42, 1.17 to 1.72), and stroke (1.55, 1.39 to 1.72) [17]. MR analysis has become a key method for etiological inference in epidemiology in recent years, as it effectively reveals causal relationships between exposures and outcomes. After a series of statistical analyses, we found that insomnia and MI have a potential causal relationship at the genetic level. Our MR analysis suggests a weak but statistically significant positive association between genetically predicted insomnia and MI risk (IVW OR = 1.015). While the effect size is modest, this finding may hold public health relevance. Given the high global prevalence of insomnia (10%–25%), even a minimal increase in individual risk could translate into a substantial population‐level disease burden [18, 19].

Coronary vulnerable plaques are defined as atherosclerotic plaques with a tendency to thrombus formation or rapid progression to culprit plaques [20]. Rapid progression and plaque rupture of vulnerable plaques leading to acute thrombus formation are the main causes of acute adverse cardiovascular events [21, 22]. Plaque ruptures predominantly occur amongst lesions characterized as TCFA [23]. Studies have shown that TCFA is an independent predictor of the composite endpoint of cardiac death, nonfatal myocardial infarction related to non‐culprit lesions, and unplanned coronary revascularization [24]. Therefore, TCFA is considered a major determinant of vulnerable atherosclerotic plaques [25]. OCT can quantitatively analyze the size of the thin fibrous cap and lipid core of vulnerable plaques, and is currently the most accurate means of assessing TCFA [6]. Patients with insomnia demonstrated significantly higher rates of TCFA, plaque rupture, and MI compared to non‐insomnia patients. While these associations are notable, establishing a definitive causal link requires further investigation. Our findings suggest that insomnia may contribute to TCFA development, potentially elevating the risk of subsequent MI.

Although the precise mechanisms linking insomnia to CVD remain incompletely understood, multiple interrelated pathways have been proposed. These include dysregulation of the hypothalamic‐pituitary‐adrenal (HPA) axis, overactivation of the sympathetic nervous system (SNS), autonomic imbalance, systemic inflammation, and accelerated atherosclerosis progression [26]. Among them, inflammation appears to play a central role. It can activate immune cells such as macrophages and T cells, which in turn release proteolytic enzymes that degrade the extracellular matrix, compromising the integrity of the fibrous cap and promoting plaque rupture [27, 28]. Experimental and clinical studies have further shown that long‐term sleep deprivation may elevate pro‐inflammatory mediators such as interleukin‐17 (IL‐17) and CRP, thereby increasing cardiovascular risk [29]. In addition, insomnia has been associated with impaired endothelial function independent of age and metabolic status, indicating that vascular dysfunction may be a key intermediary between poor sleep and increased cardiovascular risk [30].

Epidemiological data also support this link. A cross‐sectional study of 8,017 individuals found that while insomnia was not significantly associated with metabolic syndrome overall, significant associations emerged in specific subgroups. For instance, middle‐aged individuals with insomnia had a 40% increased risk of metabolic syndrome (OR 1.40, 95% CI 1.09–1.79), and men had a 35% increased risk (OR 1.35, 95% CI 1.02–1.77). Moreover, insomnia was independently associated with elevated blood pressure (24% higher odds) and reduced HDL cholesterol (16% higher odds) [31]. Notably, our study also observed increased macrophage infiltration in patients with insomnia, further highlighting the involvement of inflammation in plaque vulnerability. These findings collectively suggest that insomnia may promote cardiovascular pathology through both neuroendocrine and metabolic mechanisms, with inflammation serving as a common pathway that links sleep disturbance to vascular injury.

Beyond insomnia itself, elevated CRP levels also emerged as an independent predictor of TCFA in our analysis. This is consistent with previous findings indicating that CRP, as a biomarker of chronic systemic inflammation, is closely associated with plaque instability. Histopathological studies have shown that ruptured plaques are often characterized by persistent inflammatory activation, underscoring the central role of inflammation in atherogenesis and its complications [32]

Several important limitations merit consideration in interpreting our findings. First, the genetic analysis relied on European‐derived GWAS data while the clinical cohort was Chinese, potentially limiting cross‐population applicability. Second, the single‐center observational design with moderate sample size may not fully account for potential confounders. Third, insomnia assessment through subjective questionnaires rather than objective measures could affect accuracy. Additionally, the absence of Asian‐specific genetic data for insomnia represents a significant gap. Most importantly, while we observed associations between insomnia and plaque vulnerability, the precise pathophysiological mechanisms require elucidation through further investigation. These limitations highlight the need for future multicenter studies incorporating larger, more diverse populations, objective sleep measurements, and population‐specific genetic data to confirm and extend our findings.

## 5 Conclusion

This study provides the first dual‐dimensional evidence—combining observational OCT data and MR analysis—to suggest that insomnia may contribute to coronary vulnerable plaque formation and elevate MI risk. These findings underscore the potential value of integrating sleep management into secondary prevention strategies for CAD. Future multi‐center cohorts, transethnic MR validation, and mechanistic investigations are essential to confirm the cardiovascular benefits of insomnia interventions.

## Ethics Statement

Following the Declaration of Helsinki (as revised in 2013), this study was approved by the Medical Ethics Committee of Central China Fuwai Hospital of Zhengzhou University. Informed consent was obtained from all patients, and the data collected were kept confidential, in accordance with ethical standards.

## Conflicts of Interest

The authors declare no conflicts of interest.

## Data Availability

The datasets generated during and/or analysed during the current study are available from the corresponding author on reasonable request.
